# New Insights into Rabbit Viral Diseases

**DOI:** 10.3390/v16101521

**Published:** 2024-09-26

**Authors:** Pedro J. Esteves, Joana Abrantes, Ana M. Lopes

**Affiliations:** 1 CIBIO, Centro de Investigação em Biodiversidade e Recursos Genéticos, InBIO Laboratório Associado, Campus de Vairão, Universidade do Porto, 4485-661 Vairão, Portugal; pjesteves@cibio.up.pt (P.J.E.); jabrantes@cibio.up.pt (J.A.); 2 BIOPOLIS Program in Genomics, Biodiversity and Land Planning, CIBIO, Campus de Vairão, Universidade do Porto, 4485-661 Vairão, Portugal; 3 Departamento de Biologia, Faculdade de Ciências, Universidade do Porto, 4099-002 Porto, Portugal; 4 UMIB-Unit for Multidisciplinary Research in Biomedicine, ICBAS-School of Medicine and Biomedical Sciences, University of Porto, 4050-313 Porto, Portugal; 5 ITR-Laboratory for Integrative and Translational Research in Population Health, 4050-600 Porto, Portugal

Viruses are responsible for many devastating rabbit diseases that impact their health and welfare and put their conservation and economic revenue at risk. Myxoma virus (MYXV) has fatally infected rabbit populations worldwide since the 1950s, causing myxomatosis. Rabbit hemorrhagic disease virus (RHDV) was first described in the 1980s in China, and causes a fulminant liver disease, the rabbit hemorrhagic disease (RHD; [1]). For decades, RHDV and MYXV were specific to the European rabbit (*Oryctolagus cuniculus*). However, in 2010, a new RHDV genotype emerged [2] with an expanded host range, since it began infecting several hare species in Europe [3,4] and American leporids like jackrabbits (*Lepus* spp.), cottontails (*Sylvilagus* spp.), and pigmy rabbit (*Brachylagus idahoensis*) [5,6]. In 2018, a MYXV strain underwent recombination with an unidentified poxvirus and began to fatally infect Iberian hares (*Lepus granatensis*) [7,8]. In this Special Issue, we gathered up-to-date research on MYXV and RHDV pathogenesis. A total of seven original studies were included, all contributing to the overall knowledge of rabbit viral diseases.

The role of the European rabbit in the ecosystem is paradoxical. In the Mediterranean region, the rabbit is a keystone species, being the prey of top predators, such as the Spanish imperial eagle (*Aquila adalberti*) and the Iberian lynx (*Lynx pardinus*). It is also an iconic small game species, with hunting generating a high revenue, especially in Southern Europe. On the contrary, in countries such as Australia, New Zealand, and Chile, rabbits were introduced and are a destructive pest species, being responsible for significant damage to agriculture and ecosystems (e.g., by facilitating dispersal of exotic weeds and by competing for resources with native species) [9]. In this context, in combination with conventional control methods (for example, warren ripping), RHDV is used as a biocontrol agent in Australia and New Zealand. However, alongside the pathogenic forms, non-pathogenic lagoviruses have also been found circulating in rabbits across Europe and Oceania [10,11]. The latter confer partial immunological protection against the former, interfering with their efficiency as a form of biocontrol agent. Dorji and colleagues compared two new methods to more efficiently spread the virus with those already put in place by landowners [12], which rely on rabbits’ inoculation or actively consumed baits. These novel methods include meat bait and a soil burrow spray, both aiming to mimic natural conditions, i.e., transmission via flies and rabbit grooming behavior. While not increasing survival of the virus in the environment, both methods proved to be efficient in delivering RHDV. The authors thus recommend similar trials in wild rabbits to confirm the viability of these novel methods for population management.

Following its emergence in 2010, RHDV2 (*Lagovirus europaeus*/GI.2 [13]) observed an exceptional spread in five continents and in several lagomorph species, becoming endemic and rapidly replacing previously dominant strains. We learned that RHDV2 has been circulating in New Zealand since 2017, in another example of a rapid spread among rabbit populations. From a single incursion event, this genotype became established in the country [14]. From phylogenetic and phylogeographic analyses, the authors were able to establish that New Zealand strains arrived in the country in mid-2016 and are GI.3P-GI.2 recombinants (according to the nomenclature proposed in [13]); thus, despite their proximity, they are of non-Australian origin. The results from this study shall be considered to make informed decisions on the biocontrol of rabbit populations that do not jeopardize efforts to decrease their numbers.

Smertina and colleagues found a non-pathogenic strain in duodenum rabbit samples from Chile [15]. The Chilean non-pathogenic strain represents a novel variant (GI.4f) that probably originated from Europe with divergence set in the mid-1970s [15]. This study warrants new information on naturally circulating lagoviruses and uncovers important information on the evolutionary mechanisms of these viruses, as evolution from pre-existing non-pathogenic strains has been postulated as a mechanism for virulence acquisition [16]. Finally, this work allowed to sequence the entire genome of a rabbit astrovirus from an apparently healthy animal. Astroviruses cause gastroenteritis, diarrhea, and dehydration, particularly in young rabbits, and co-infection with other viruses might increase the likelihood of rabbits developing enteric disease. The implications of this finding should be investigated, as cross-species transmission between wild animals, humans, and livestock has raised concerns about their zoonotic potential in recent years (e.g., [17]).

We also learned about the benefits of actively involving society in research [18]. Citizen science is being applied in several areas of knowledge (e.g., [19,20]), greatly complementing knowledge retrieved from research alone in a cost-efficient manner. The lack of a sufficient number of samples to provide a robust understanding of lagovirus epidemiology led Australian researchers to implement a citizen science program, RabbitScan (https://www.feralscan.org.au/rabbitscan/, accessed on 17 September 2024) [18]. This program provides members of the public with submission kits and instructions for collecting and storing samples from deceased leporids for lagovirus testing. Putting this program into practice allowed to follow lagoviruses’ distribution and genetic diversity, including the detection of recombinant strains. Notably, this method proved that domestic animals serve as a proxy and are “good indicators of the natural circulation of lagoviruses in the wild”. We encourage other researchers to apply similar methodologies to better control the disease, accompanied by a strong engagement program, namely in Europe and North America, where RHDV2 is widespread and has an expanded host range.

Myxomatosis is another important disease of leporids, primarily affecting the European rabbit. It was deliberately introduced in Europe in the 1950s as a biocontrol agent and, since then, it spread among domestic and wild rabbit populations [1]. A few instances of host jumps to European and mountain hares (*L. europaeus* and *L. timidus*, respectively) have been reported, but with no associated outbreaks [21]. Since 2018, a new strain, MYXV-Tol or MYXV-ha, disseminated among Iberian hare populations (*L. granatensis*). In this Special Issue, a case of myxomatosis in an Italian hare (*L. corsicanus*) with lesions suggestive of the disease was confirmed with successful viral isolation and electron microscopy identification [22]. The characterization of the genome of this MYXV strain further revealed several mutations in genes important to host range tropism or virulence, confirming that differences in these genes impact poxviruses’ host range [23]. Interestingly, this hare was also infected by *Treponema* spp., a sexually transmitted bacteria causing syphilis, which might have favored MYXV infection into a new species. This work highlights the importance of population monitoring and epidemiological studies for successful wildlife conservation.

Domestic rabbits are also of note, either as companion animals or in rabbit meat and fur production. Although preventing disease is not possible in the wild, there are licensed vaccines worldwide that can reduce the burden of RHD in domestic animals. Vaccine production relies on several methods, from inactivated viral vaccines to those based on virus-like particles or recombinant viral vectors. Recombinant vaccines are safer and more efficient. In this Special Issue, we were informed of the development of a baculovirus recombinant vaccine against RHDV2 that is capable of eliciting strong humoral and cell-mediated immunity in rabbits six months after vaccination [24]. Moreover, booster vaccination dramatically increases titers, leading the authors to recommend rabbit owners to consider this scheduling scheme to mitigate the risks associated with RHD outbreaks. This new vaccine expands the current prophylactic measures available to tackle RHDV2.

Of equal importance is the understanding of the host cellular mechanisms triggered after viral infection. Indeed, it has been shown that susceptibility to infection and clinical outcomes differ in immunocompromised host individuals [25,26]. The spleen is a key organ in this process, as it contains lymphocytes and phagocytes that mediate the humoral and cellular response. Yu and colleagues presented an RNA-seq analyses of the splenic transcriptome after RHDV2 challenge, showing the downregulation of genes involved in host defense and the upregulation of genes associated with disease, signal transduction, cellular processes, and cytokine signaling [27], resulting in chronic inflammation and exacerbated immune responses. These findings are crucial for a better understanding of RHDV2 pathogenicity and of the role of the spleen in virus–host interactions.

With this, we would like to acknowledge all authors and co-authors for providing a fascinating snapshot of current research on rabbit viral diseases. It has been a pleasure to guest this Special Issue and learn from all the contributions. Together, we must continue our efforts on disclosing and understanding the distribution, evolution, and mechanisms that determine the host range and pathogenicity of viruses affecting rabbits, to help reduce their impact on domestic and wild rabbits and other leporid species. Furthermore, this knowledge can pave the way for using leporids as animal models to study viral diseases. We hope that this subject will assist scientists in the development of efficient antivirals and serve as a substantial source of knowledge and inspiration for researchers, students, and stakeholders.
